# Syphilis of the Eye

**Published:** 1904-08-13

**Authors:** 


### SYPHILIS OF THE EYE.

Jessop [1] classifies syphilitic affections of the eye into congenital and acquired. The latter are met with in all three stages. The primary affection is sometimes seen on the lids or conjunctiva, but diagnosis must remain uncertain until secondary symptoms show themselves. Iritis and choroiditis are the forms in which secondary syphilis usually appears in the eye, and these lesions are liable to ensue in from two to nine months from the time of infection. Tertiary manifestations are pigmentary retinitis, choroido-retinitis, irido-cyclitis, affections of the optic nerve, parenchymatous keratitis, ulcerations of the lids, periostitis and necrosis of the orbital bones, and paralyses of muscles—extrinsic and intrinsic. Treatment should be local and general. General treatment should consist of administration of mercury or potassium iodide—according to the stage. The mercury for its effect to be permanent should be given in small doses over a long time, although immediate good results are got from large doses. It is best administered by inunction. In addition a piece of the half strength unguentum hydrargyri (B.P.) of about the size of a hazel nut can be rubbed in over the affected eye each night with advantage. Darier [2] has recommended subconjunctival injection. To do this 2 per cent. cocaine hydrochloride should be instilled into the conjunctival sac five minutes before injecting, then a drop or two of 1 per cent. solution of cocaine should be mixed with 6 minims of a solution of 1 in 1000 cyanide of mercury. The needle should be passed beneath the subfascial tissue, and the injection reach behind the equator into the retro-bulbar tissue. In interstitial keratitis subconjunctival injection of 6 minims of 5 per cent. sodium chloride solution is followed by good results. At times the results of mercurial treatment are at first small (even for more than a year), but persistence will bring its reward. The local treatment must vary with the part affected. A primary sore must be kept clean, and have a weak mercurial ointment or lotion applied to it. Iritis must be treated with a mydriatic so as to keep the pupil at rest, and prevent the formation of posterior synechiæ; the eyeball must be kept warm and at rest to relieve pain, and the tendency to vascular congestion. The best mydriatic is atropine, either in the form of drops (2 to 4 grs. to 1 oz.), ointment (1 gr. to 1 oz.), or disc ($\frac{1}{250}$ gr.). Its action is assisted by the use of cocaine. It is well to make three or four applications at intervals of a few minutes at the commencement, and to continue with three or four a day. Rest for the eyeball can be obtained by a firm bandage, and the sound eye ought to be shaded from the light. After the bandage has been removed goggles should be worn, with cotton wool placed in the eyepiece of the affected eye. Then neutral-tinted or blue spectacles may take the place of the goggles. Leeching of the temporal region will relieve congestion. Dionine (5 per cent.) instilled into the eye often produces marked relief of pain, its first effect is to produce chemosis and its analgesic action comes on in about two hours. In retinitis or choroiditis tinted glasses ought to be worn; and atropine may be used to keep the eye at rest. Local treatment of paralysis and pareses of muscles must be directed to stimulate action, *e.g.* pilocarpine and eserine for the intrinsic muscles, and exercises and electricity for the extrinsic muscles. Tertiary lid affections ought to be treated with mercurial ointments. Periostitis and necrosis of the orbit should be met with local sedative applications and the removal of dead bone. Interstitial keratitis during the acute stage must be treated with atropine, and in the subacute with an ointment of yellow oxide of mercury 2 to 8 gr., atropine 1 gr., vaseline 1 oz.

[1] Practitioner, July 1904. [2] Ocular Therapeutics, Darier and Stephenson, 1903, p. 28.

